# Optimization of Ultrasonic-Assisted Simultaneous Extraction of Three Active Compounds from the Fruits of *Forsythia suspensa* and Comparison with Conventional Extraction Methods

**DOI:** 10.3390/molecules23092115

**Published:** 2018-08-23

**Authors:** Xinsheng Fang, Shubo Gu, Zongyuan Jin, Mingqian Hao, Zhenzhen Yin, Jianhua Wang

**Affiliations:** State Key Laboratory of Crop Biology, Shandong Key Laboratory of Crop Biology, College of Agronomy, Shandong Agricultural University, Taian 271018, China; gusb@sdau.edu.cn (S.G.); 17863801898@163.com (Z.J.); h74596170@163.com (M.H.); genuinecm@sina.com (Z.Y.)

**Keywords:** ultrasonic-assisted extraction, *Forsythia suspensa*, active compounds, response surface methodology

## Abstract

An efficient ultrasonic-assisted extraction (UAE) method was developed for simultaneous extraction of three active compounds, forsythiaside A (FSA), phillyrin (PHI) and rutin (RT), from the fruits of *Forsythia suspensa*. The effects of various factors including a binary mixed solvent of methanol/water and ethanol/water, the pH of the solvent, particle size, temperature, solvent to material ratio, ultrasonic input power and extraction time on UAE were investigated in detail. The mass transfer mechanism of UAE using different mixed solvents was further explained by comparison with the maceration extraction method. The response surface methodology was used to optimize the experimental variables including ethanol concentration, solvent to material ratio and extraction time. The optimized conditions for the simultaneous extraction of RT, FSA and PHI were: particle size 60–80 mesh, temperature 30 °C, ultrasonic power 200 W, ethanol concentration 50%, solvent to material ratio 32 mL/g and extraction time 37 min. Compared to conventional extraction methods, UAE provided the highest extraction efficiency and offered many advantages including the reduction of solvent, temperature and time for extraction.

## 1. Introduction

**Figure 1:**
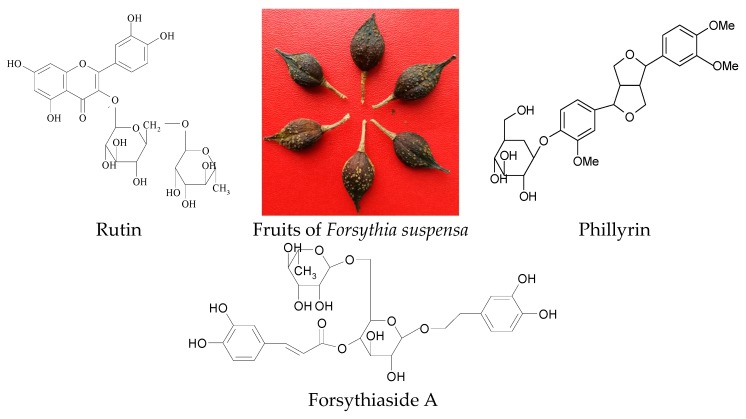
The fruits of *Forsythia suspensa* and the chemical structures of rutin, forsythiaside A and phillyrin.

*Forsythia suspensa* (Thunb.) Vahl. is widely distributed in China, Korea, Japan and many European nations. The fruit of *F. suspensa* (Lianqiao in Chinese, weeping forsythia in English) is a well-known traditional Chinese medicine [1]. Lianqiao has been widely used as an antipyretic and antidotal agent and has shown the effects of being anti-inflammatory, antibacterial, antiviral, antioxidant, resisting hepatic injury, etc. [2,3,4]. More than 40 Chinese medicinal preparations containing Lianqiao are recorded in the Chinese Pharmacopoeia, such as Shuanghuanglian granule, Yinqiao Jiedu tablet, Qinlian tablet, etc. [1]. Phytochemical studies have shown that forsythiaside A (FSA), phillyrin (PHI) and rutin (RT) are all the main bioactive components in Lianqiao [5,6,7,8]. Forsythiaside A was reported to have various biological activities such as antibacterial [2], antiviral [9], antioxidant [10], vasorelaxant [11] and a neuroprotective effect [12,13]. Phillyrin showed the activities of being antibacterial [5], antivirus [14] and having a protective effect on LPS-induced acute lung injury [15]. Rutin also has various activities that are beneficial to human health such as an antioxidant effect [16], a protective effect against hepatotoxicity [17], vasoprotective activity [18], anti-inflammatory [19] and neuroprotective effect [20]. The structures of the three compounds and the picture of Lianqiao are shown in Figure 1.

Ultrasonic-assisted extraction (UAE) is an efficient technology that has been attracting much more attention in separation and extraction in recent years. It has shown many benefits such as lower solvent consumption, lower energy input, lower temperature and faster extraction rate in the extraction of plant active compounds [21]. UAE has been used in the extraction of flavonoids [22], essential oils [23], phenylpropanoids [24], phenolic compounds [25,26], antioxidants [27,28], etc. In conventional extraction methods free diffusion is the main extraction mechanism, which can be greatly influenced by the polarity of the solvent and target compound. However, in UAE, the cavitation effect is the main factor, which creates shear forces that break cell walls mechanically and improves mass transfer [29,30]. The properties of the solvent such as density, viscosity, surface tension and vapor pressure could influence the ultrasound effect and therefore affect the extraction efficiency [21]. The application of mixed solvents, rather than a single solvent in UAE would provide appropriate properties for extraction. Methanol (or ethanol) and water are the conventional solvents for extraction. A comprehensive investigation of the effect of mixed solvent systems of methanol/water and ethanol/water on UAE may improve the efficiency of UAE and economize the use of energy, organic solvents and time. Up to now, the mechanism of extracting active compounds from herbals by UAE is still under study.

The conventional methods to extract the three compounds from Lianqiao are heat reflux extraction (HRE) [31] and decocting extraction (DE) [1]. In the previous study, the extraction of forsythosides A and forsythosides I from the leaf of *F. suspense* using ionic liquid-based UAE was developed for their following purification using high-speed counter-current chromatography [32]. In view of the important bioactivities of FSA, PHI and RT, the efficient extraction of the three compounds from Lianqiao was essential for their application in medicine. The extraction of forsythiaside A, rutin and phillyrin using microwave-assisted extraction has been developed [33]. The objectives of this study were to develop an ultrasonic-assisted extraction method for the three compounds in Lianqiao, investigate the effect of various factors on UAE and provide more information to understand the mechanism of UAE from natural products. The dissolving out characteristics of the three compounds using UAE were investigated by comparison with the extraction method of maceration. The efficient UAE process for the simultaneous extraction of FSA, PHI and RT was optimized using the response surface methodology (RSM) for high recovery and avoiding degradation of the components. In addition, the efficiency of the developed UAE was also compared with three conventional extraction methods.

## 2. Results and Discussion

### 2.1. Effect of a Single Factor on Extraction Yields

#### 2.1.1. Effect of Solvent and Comparison with Maceration

**Figure 2:**
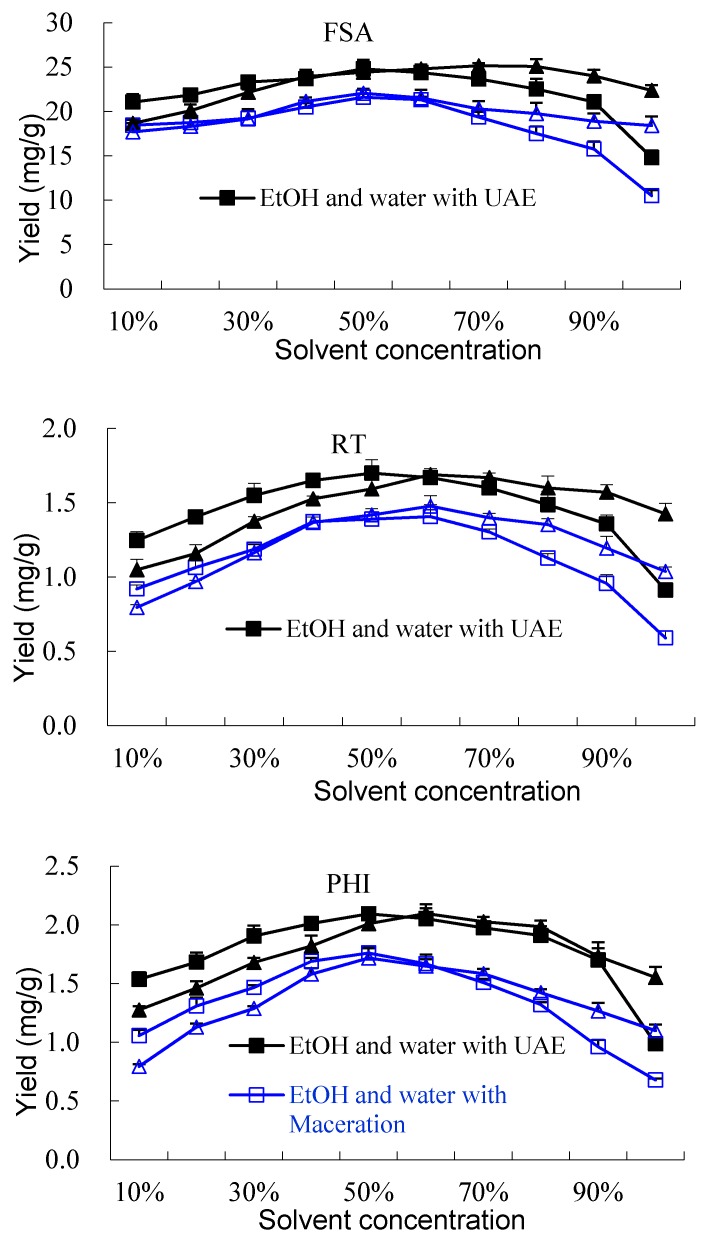
Effect of different solvents on the extraction yields of forsythiaside A (FSA), rutin (RT) and phillyrin (PHI). UAE, ultrasonic-assisted extraction.

The effects of the mixed solvent systems of methanol/water and ethanol/water were firstly studied, which would help to find the most suitable solvent for the efficient extraction and discuss the mechanism of UAE. One gram of powder (40–60 mesh) was extracted with 20 mL of different mixed solvents (10–100% (*v*/*v*) aqueous methanol and 10–100% (*v*/*v*) aqueous ethanol) for 10 min. The input power was 200 W. The temperature was 25 °C. The yields of the three components using different mixed solvents are shown in Figure 2. The yields of the three components showed different variation trends between the solvent of 10–100% methanol and 10–100% ethanol. The yields of the three components increased significantly (*p* < 0.05) when the solvent changed from 10–40% aqueous ethanol and reached the maximum when the solvent was 50% ethanol. The yields showed a downward trend when the solvent changed from 60–100% ethanol. However, when the solvent was the mixture of methanol/water, the yields of RT, FSA and PHI increased when the solvent changed from 10–70% methanol and showed not significant difference (*p* > 0.05) when the solvents were 50–80% methanol. The yields of FSA and RT using 90–100% methanol were significantly higher than using 90–100% ethanol. The yield variation of the three components presented the form of a logarithmic curve when the solvent changed from 10–80% methanol; while when the solvent changed from 10–100% ethanol, the variation trends presented a parabolic curve that could be fit as quadric polynomial models with a good determinant coefficient. The difference of the yield between the mixture of ethanol/water and methanol/water may be due to the different properties of the mixed solvents such as polarity, surface tension, critical molecular distance, vapor pressure and viscosity [27,29,34]. In UAE, the critical molecular distance is closely related to the production of cavitation bubbles, which are responsive to the ultrasonic effect. When methanol or ethanol was mixed with water in different proportions, different molecular distances existed in the mixed solvent. In addition, the surface tension, vapor pressure and viscosity of the mixed solvent were also changed. Therefore, the cavitational effect of the mixed solvent would be changed. The suitable proportion of water with methanol (or ethanol) would improve the extraction efficiency because of the enhanced cavitational effect of the mixed solvent and suitable solubility of the components in the solvent [21,26]

As mentioned above, the cavitational effect is the primary extraction mechanism, which facilitates the release of extractable compounds and enhances the mass transport [34,35]. In conventional extraction methods, free diffusion is the main extraction mechanism. The solubility of the target components in solvent was an important factor. By comparing with maceration, the dissolution mechanism of the components using UAE can be further explained. The extraction yields of the three components using maceration with different mixed solvents are shown in Figure 2. When using maceration, the extraction efficiency was mainly affected by the solubility of the components in solvent and the permeation of the solvent into the matrix. The results indicated that 40–60% ethanol and 50–60% methanol were most suitable for the dissolution and diffusion of FSA; 50–60% ethanol and 50–60% methanol were most suitable for RT; 40–50% ethanol and 50–60% methanol were suitable for PHI. The yields of UAE were always higher than those of maceration when using the same mixed solvent. The higher yield of UAE was mainly due to the ultrasonic cavitational effect. The variation trends of the yields of UAE were similar to those of maceration when the solvents were 10–50% methanol (or ethanol). However, when the solvents were 60–90% methanol (or ethanol), the yields of maceration were significantly less than those of UAE, which indicated that ultrasonic cavitation showed a more important effect than free diffusion of compounds in these solvents. 

The above discussions indicated that the suitable mixed proportion of water with methanol (or ethanol) will improve the extraction efficiency because of the enhanced cavitation effect and mass transfer. The highest yields of RT, FSA and PHI using UAE can be simultaneously obtained by 50–60% aqueous ethanol or 60–80% aqueous methanol. Considering the toxicity and safety, 50% aqueous ethanol was chosen as the solvent for the extraction of the three components in the following one-factor experiments. 

#### 2.1.2. Effect of pH of the Solvent

**Figure 3:**
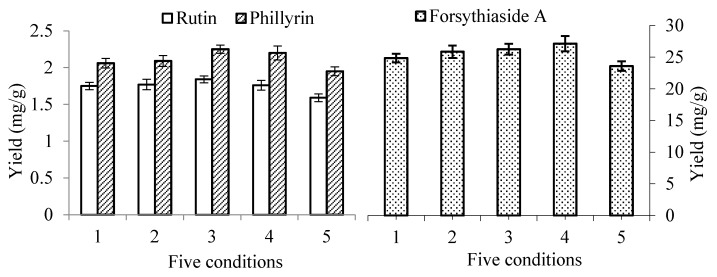
Effect of different conditions on the extraction yields of forsythiaside A, rutin and phillyrin when acetic acid was added to the solvent. The value of forsythiaside A is shown on the secondary *y*-axis on the right. Condition 1: 50% ethanol as solvent, ultrasonic power 200 W, temperature 30 °C; Condition 2: 50% ethanol + 2% acetic acid as solvent, ultrasonic power 200 W, temperature 30 °C; Condition 3: 50% ethanol + 5% acetic acid as solvent, ultrasonic power 200 W, temperature 30 °C; Condition 4: 50% ethanol + 5% acetic acid as solvent, ultrasonic power 200 W, temperature 50 °C; Condition 5: 50% ethanol + 5% acetic acid as solvent, ultrasonic power 100 W, temperature 30 °C.

In order to study the pH of the solvent on the extraction efficiency of UAE, different amounts of acetic acid (2% and 5%, *v*/*v*) were added to 50% aqueous ethanol used as the solvent. An amount of 1 g powder (40–60 mesh) was extracted with 20 mL mixed solvent. The extraction time was 10 min. In addition, the efficiency under different ultrasonic input powers and different temperatures using 50% ethanol containing 5% acetic acid was also studied. The results are shown in Figure 3. The yields of the three compounds increased when 2% and 5% acetic acid were added to 50% ethanol. The yields of RT and FSA showed no significant increase (*p* > 0.05) when acetic acid was added. However, the yields of PHI increased significantly (*p* < 0.05) when 5% acetic acid was added. Between conditions 3 and 4, when the temperature changed from 30–50 °C, the yields of RT and PHI decreased slightly, but the yields of FSA increased slightly. Between Conditions 3 and 5, when the ultrasonic power changed from 200–100 W, the yields of the three compounds all decreased significantly (*p* < 0.05). The results indicated that adding 5% acetic acid to the solvent was not suitable for simultaneously improving the extraction efficiency of the three components. Furthermore, adding acetic acid to the solvent would influence the determination and separation of the compounds. Therefore, only 50% ethanol was used for the solvent in the following experiments.

#### 2.1.3. Effect of Particle Size of the Sample

**Figure 4:**
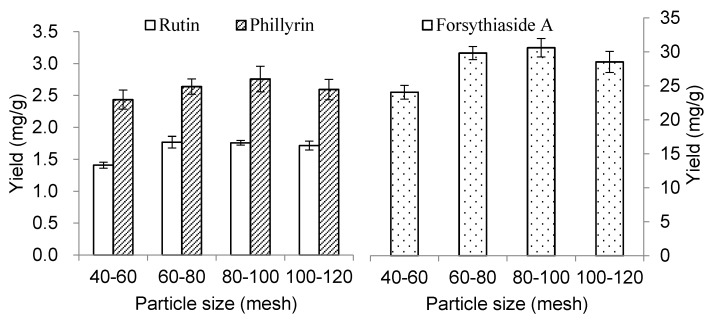
Effect of particle size on the extraction yields of forsythiaside A, rutin and phillyrin. The value of forsythiaside A is shown on the secondary *y*-axis on the right.

An amount of 1 g powder was extracted with 30 mL 50% aqueous ethanol under different particle sizes (40–60, 60–80, 80–100 and 100–120 mesh). The ultrasonic input power was 200 W and extraction time 30 min. The results are shown in Figure 4. The yields of the three components in 40–60 mesh were all significantly (*p* < 0.05) lower than those in 60–80, 80–100 and 100–120 mesh. Though the yield of FSA decreased when using 100–120 mesh, the yields showed no significant difference (*p* > 0.05) among 60–80, 80–100 and 100–120 mesh for the three components. Therefore, the particle size of 60–80 mesh was used in the following experiments.

#### 2.1.4. Effect of Temperature

**Figure 5:**
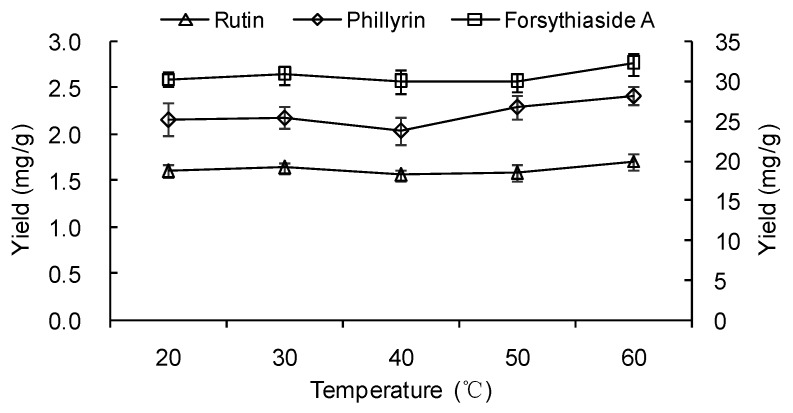
Effect of temperature on the extraction yields of forsythiaside A, phillyrin and rutin. The value of forsythiaside A is shown on the secondary *y*-axis on the right.

An amount of 1 g powder was extracted with 30 mL 50% ethanol for 30 min. The input power was 200 W. The extraction yields at different temperatures (20, 30, 40, 50 and 60 °C) were compared. The results (Figure 5) indicated that the yields of three components decreased when the temperature changed from 20–40 °C and increased from 40–60 °C. The yields of the three components at 60 °C were significantly higher (*p* < 0.05) than those at 40 °C. However, the yields showed no significant difference (*p* > 0.05) among the temperatures of 20–50 °C for the three components. In UAE, the extraction yield could be affected by the combined action of acoustic cavitation and the thermal effect. High temperature led to the decrease of the cavitation effect because of the increase of vapor pressure and the decrease of viscosity and surface tension of the solvent, which resulted in the decrease of extraction efficiency [36,37]. However, a higher temperature increased free diffusion of the components, which resulted in the increase of mass transfer. The influence of the cavitation effect and thermal effect on the yield would arrive at the equilibrium state at a certain temperature. The temperature of 40 °C would be a threshold value for high extraction yields of the three components. When the temperature rose to 60 °C, the thermal effect played a greater role in the extraction efficiency. Considering the saving of energy and easy operation, 30 °C was used as the temperature in the following experiments. 

#### 2.1.5. Effect of Solvent to Material Ratio

**Figure 6:**
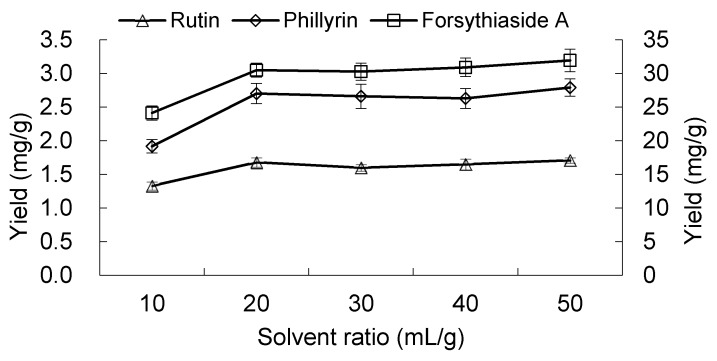
Effect of solvent to material ratio on the extraction yields of forsythiaside A, phillyrin and rutin. The value of forsythiaside A is shown on the secondary *y*-axis on the right.

An amount of 1 g powder was extracted with 50% aqueous ethanol for 30 min using different solvent to material ratios (10, 20, 30, 40 and 50 mL/g). The input power was 200 W and temperature 30 °C. The yields of the three components using a 20 mL/g solvent to material ratio were significantly higher (*p* < 0.05) than those of 10 mL/g (Figure 6). For the three components, the yields showed no significant difference (*p* > 0.05) when the solvent to material ratio changed from 20–50 mL/g. In general, a higher solvent to material ratio could cause a greater concentration difference, which facilitated the diffusion of components into solvents and accelerated mass transfer [21]. However, in UAE, the particular extraction mechanism could play a greater role in the extraction than the free diffusion mechanism [27,38,39]. Therefore, the increase of the solvent to material ratio may not lead to the increase of extraction efficiency. In the present study, a 20 mL/g solvent to material ratio would be a threshold value for the extraction of the three components when using UAE. 

#### 2.1.6. Effect of Ultrasonic Input Power and Extraction Time

**Figure 7:**
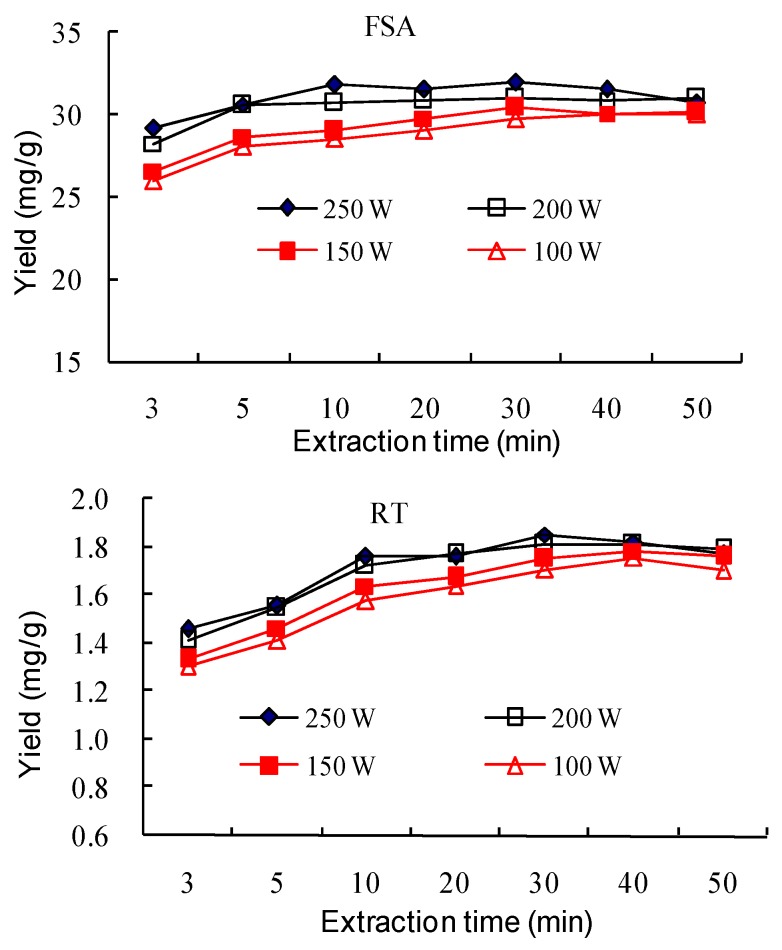

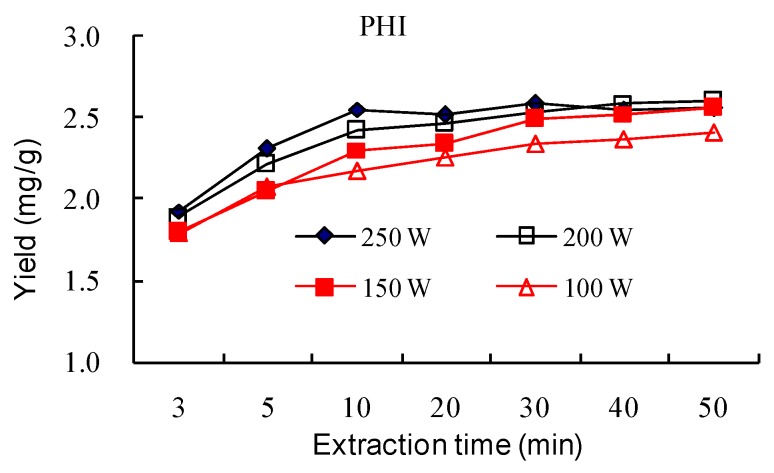
Effect of ultrasonic input power and extraction time on the extraction yields of forsythiaside A (FSA), rutin (RT) and phillyrin (PHI).

An amount of 1 g powder was extracted with 20 mL 50% aqueous ethanol for different times (3, 5, 10, 20, 30, 40 and 50 min) using different input powers (250, 200 150 and 100 W), respectively. The results are shown in Figure 7. The yields of the three components using different power increased with the time increasing from 3 to 10 min. When the power was 250 and 200 W, The yields of FSA, RT and PHI reached the maximum at 10, 30 and 30 min, respectively. However, when the input power was 150 W, the yields of FSA, RT and PHI reached the maximum at 30, 40 and 40 min, respectively. When the input power was 100 W, the yields of FSA, RT and PHI reached the maximum at 40, 40 and 50 min, respectively. The cavitation effect enhanced with the increase of ultrasound input power, which facilitated the disruption of the cell wall and the interior components’ release to the solvent. The fast increase of the yields from 1–5 min may be due to the easier diffusion of components from outer part and the large concentration gradient between the extracting solvent and the cell. However, when the mass transfer reached equilibrium, the yield would not increase with the prolonged of extraction time. 

The input power has both a positive and negative influence on the cavitation effect. On the one hand, higher ultrasonic intensity resulted in a greater number of cavitation micro-bubbles being created, and more energy was obtained when the bubbles drastically collapsed, which facilitated the disruption of tissue cell walls and accelerated mass transfer [40]. On the other hand, with the increase of acoustic intensity, more bubbles were formed, which hampered the propagation of shock waves, and the bubbles may have coalesced to form bigger ones and weakly imploded. Hence, the extraction efficiency would be decreased [39]. There would be a balance between the positive and negative effect of the input power on the extraction. In the present study, the yields of the three components using a power of 250 W were close to those of 200 W at each extraction time. The same phenomenon could be found between the power of 150 and 100 W. Therefore, the power of 200 W was used in the following optimization.

### 2.2. Optimization of UAE Using RSM

#### 2.2.1. Modeling of the Extraction Yields and Response Surface Analysis

**Figure 8:**
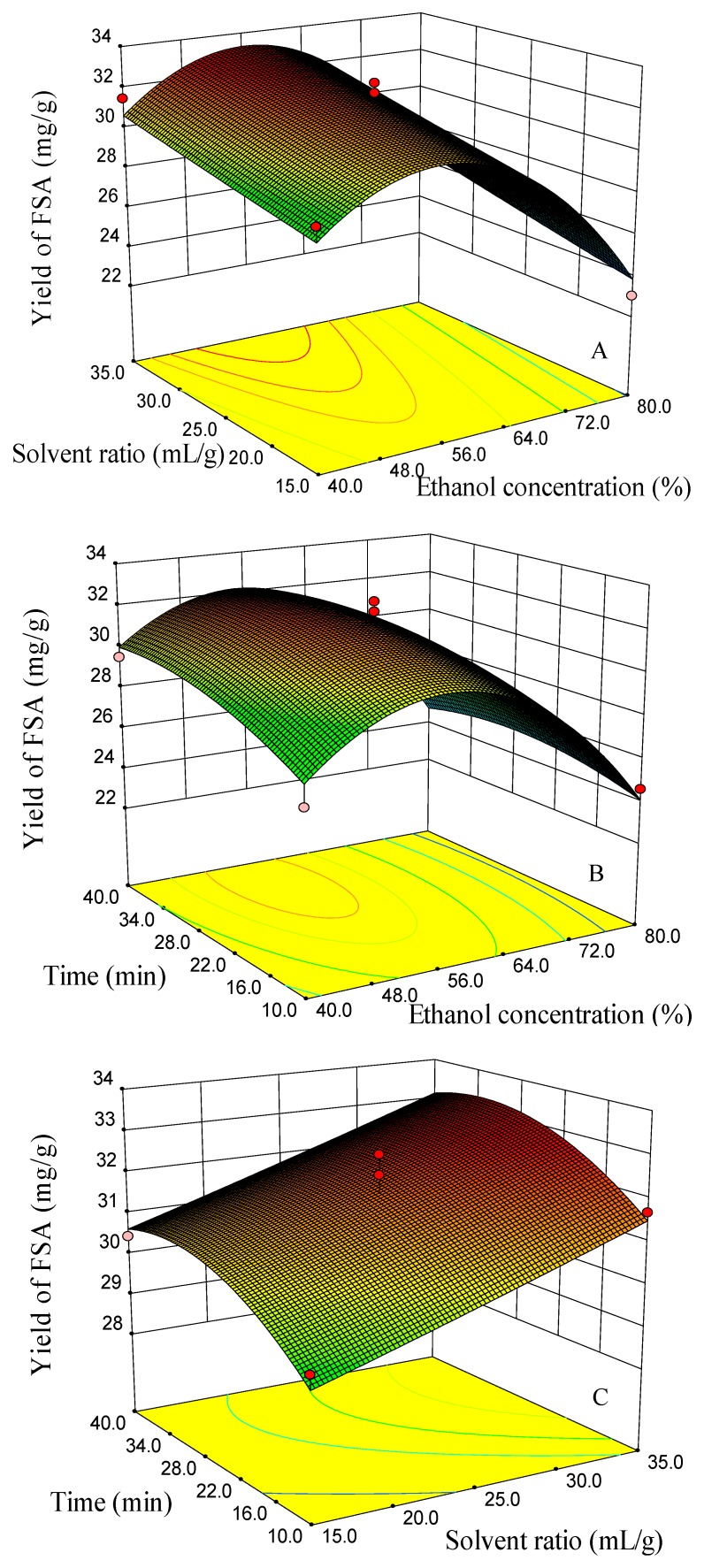
Response surface and contour plots for the effect of (**A**) EtOH concentration/solvent ratio, (**B**) EtOH concentration/time and (**C**) time/solvent ratio on the yields of forsythiaside A (FSA).

**Figure 9:**
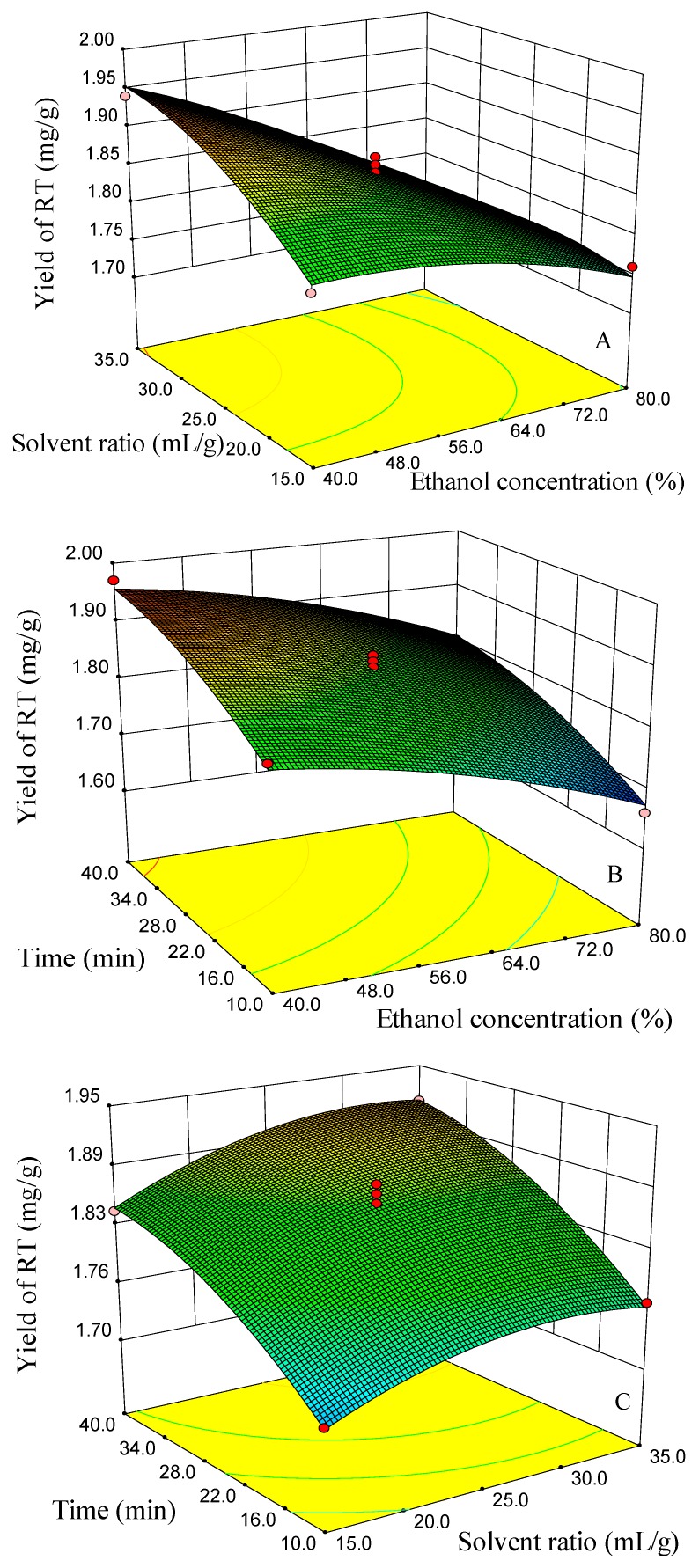
Response surface and contour plots for the effect of (**A**) EtOH concentration/solvent ratio, (**B**) EtOH concentration/time and (**C**) time/solvent ratio on the yields of rutin (RT).

**Figure 10:**
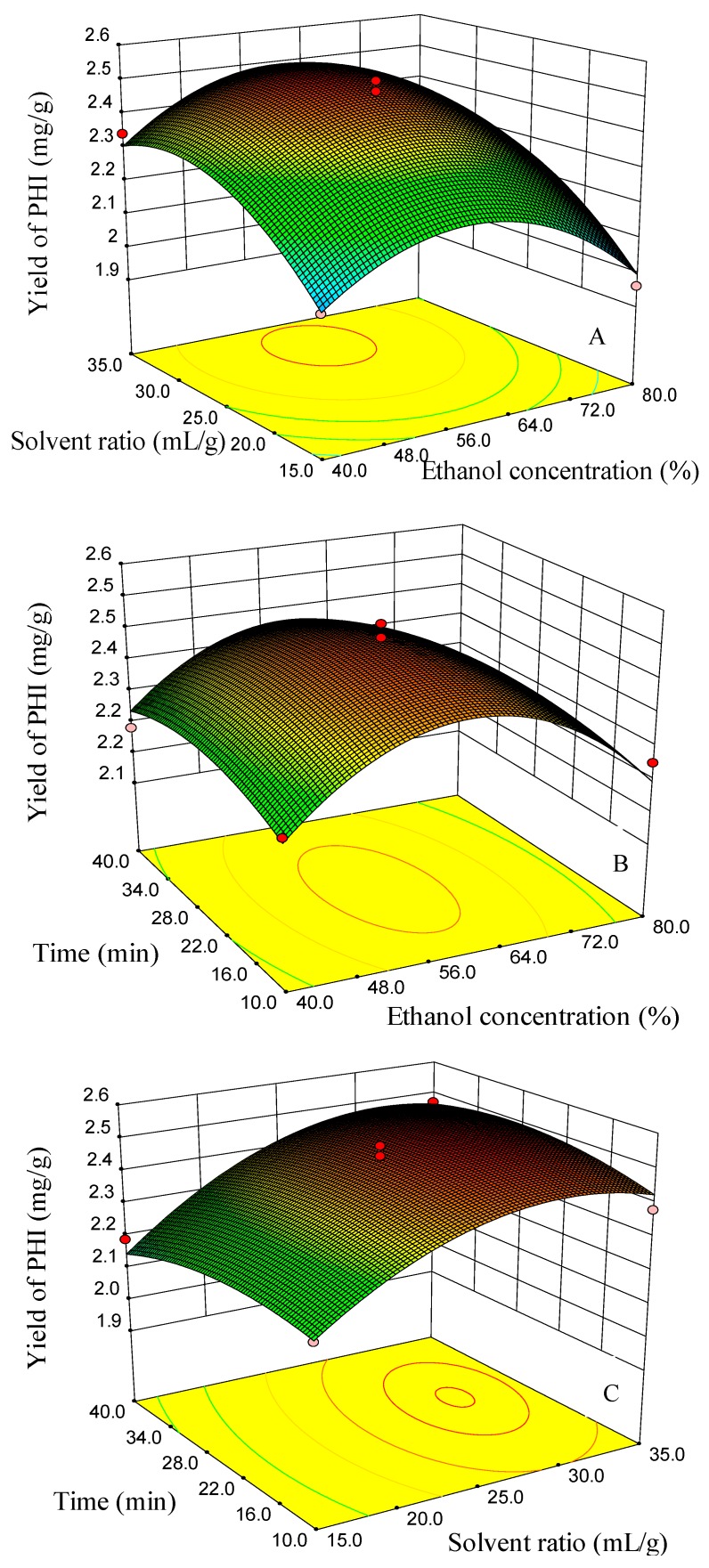
Response surface and contour plots for the effect of (**A**) EtOH concentration/solvent ratio, (**B**) EtOH concentration/time and (**C**) time/solvent ratio on the yields of phillyrin (PHI).

The Box–Behnken design and the responses measured are shown in Table 1. Regression analysis was carried out using the statistical software of Design-Expert 8.0.6. The ANOVA analysis showed that the three quadratic models were significant at 95% confidence, and the lack of fit was not significant, which indicated that the three fitted models were considered adequate. Model coefficients for the three responses are shown in Table 3. Surface response graphs, obtained using the fitted model, are presented in Figure 8, Figure 9 and Figure 10. 

The yields of FSA were at a higher level when the ethanol concentration changed from 50–60%, but showed a decreasing trend when the ethanol concentration changed from 60–80% (Figure 8A,B). When the ethanol concentration was 60%, the yields of FSA showed a slight decreasing trend with the time increasing from 35–40 min (Figure 8C). The reason may be due to the degradation of FSA because of the continuous collapse of cavitation bubbles in the ultrasound treatment [36,41]. When the solvent/material ratio was 25 mL/g, the higher yields were obtained at ethanol concentrations of 50–55% and extraction times of 25–35 min (Figure 8B). Surface response graphs of the yields of RT are presented in Figure 8. Ethanol concentration, solvent/material ratio and extraction time all showed a significant influence on the yield of RT. The effects of the solvent to material ratio and ethanol concentration on the yield of RT showed a significant interaction (Table 2). When the ethanol concentration was 40–50%, the yield of RT increased with the increasing of the solvent to material ratio. However, when the ethanol concentration was 65–80%, the yield of RT changed slightly with the increasing of the solvent to material ratio (Figure 9A). When the solvent/material ratio was 25 mL/g, the higher yields of RT were obtained using a solvent of 40–50% ethanol and a time of 35–40 min (Figure 9B). When the ethanol concentration was 60%, the higher yields of RT could be obtained using a solvent/material ratio of 25–35 mL/g and a time of 35–40 min (Figure 9C). The higher yield of PHI can be obtained when the ethanol concentration was between 50% and 60% and the solvent/material ratio between 25 and 35 mL/g with an extraction time of 25 min (Figure 10A). When the ethanol concentration was 60%, the higher yields of PHI were achieved when the extraction time was between 25 and 35 min and the solvent ratio was between 30 and 35 mL/g (Figure 10C).

#### 2.2.2. Optimization of the UAE

In this study, the aim of optimization was to find the conditions that gave the maximum yields for simultaneous extraction of FSA, RT and PHI. However, the examination of all the results obtained by the response surface analysis showed that it was not easy to determine experimental conditions that could optimize all the responses simultaneously. Taking into account this situation, we used the desirability function approach of Derringer [42] to search the experimental conditions that could optimize all the responses simultaneously. The Derringer desirability function was carried out with the software Design-Expert 8.0.6. The same importance degree was given for the three components. The software predicted the optimum conditions to be: ethanol concentration 50%, solvent to material ratio 32 mL/g and extraction time 37 min. 

### 2.3. Validation of UAE and Comparison with Conventional Methods

Based on the results of the single-factor and RSM experiments, the optimum conditions for simultaneous extraction of FSA, PHI and RT were: particle size 60–80 mesh, temperature 30 °C, ultrasonic power 200 W, solvent 50% ethanol, solvent to material ratio 32 mL/g and extraction time 37 min. The optimized conditions of UAE were verified by three parallel experiments. The extraction yields of FSA, PHI and RT are presented in Table 3. The predicted results matched well with the experimental results obtained using the optimum extraction conditions, which indicated that the optimization was reliable in this study. 

In addition, to evaluate the extraction efficiency of the developed UAE, the conventional extraction method of HRE, Soxhlet extraction (SE) and DE were also applied for the extraction of the three components from Lianqiao (Table 3). The extraction yields of the three compounds with the developed UAE were significantly higher (*p* < 0.05) than those of HRE in 90 min. The yields of FSA showed no significant difference (*p* > 0.05) between SE and UAE. However, the yields of PHI and RT using SE were significantly lower (*p* < 0.05) than those of UAE. DE was the method used for the preparation of Lianqiao extract in the Chinese Pharmacopoeia. The results indicated that the yields of the three components were very low when using DE for the extraction of active compounds from Lianqiao. Therefore, the higher yields of FSA, RT and PHI cannot be simultaneously obtained when using the conventional extraction methods. However, when using the developed UAE, the highest yields of the three compounds can be simultaneously obtained in 37 min.

### 2.4. Analytical Method Performance

**Figure 11:**
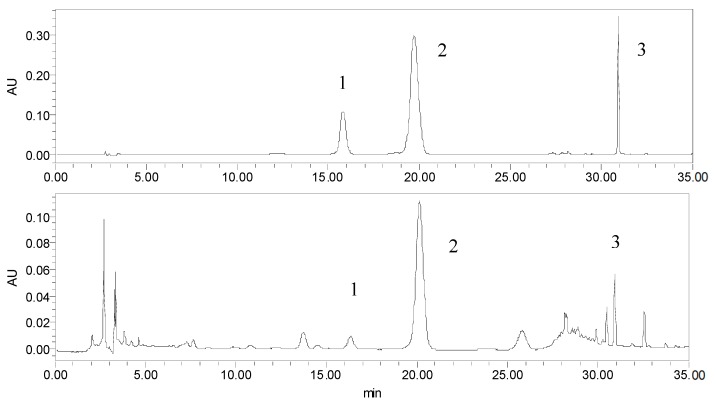
The HPLC chromatograms of the standards and sample (278 nm) obtained by UAE (1. rutin, 2. forsythiaside A, 3. phillyrin).

The HPLC chromatograms of the standards and sample obtained by UAE are shown in Figure 11. The chromatographic method presents a good linearity in the concentration range considered. The correlation coefficient and linear range of the three compounds are listed in Table 4. The intraday precision was determined by analyzing in triplicate the same solution five times within one day; while for inter-day variability test, the solution was examined in triplicate for three consecutive days. The results showed that the RSD of intra-day variability were 1.22%, 0.57% and 2.06% for RT, FSA and PHI, respectively; while the inter-day precision was 2.90%, 3.15% and 3.37% for RT, FSA and PHI, respectively. The recoveries of the three compounds were 95.9%, 98.4% and 103.6%, respectively. According to the results of linearity, precision and recovery test, it was known that the chromatographic method was suitable for the determination of the three compounds.

## 3. Materials and Methods

### 3.1. Materials, Standards and Reagents

The fruits of *F. suspensa* were collected from Shandong in China. The fruits were dried in the drying room with active ventilation at room temperature until constant weight. Standard substance of forsythiaside A, phillyrin and rutin were obtained from the National Institute for the Control of Pharmaceuticals and Biological Products (Beijing, China); the purity of the standards was above 98% (*w*/*w*). Phosphoric acid, methanol, ethanol (analytical grade) and acetonitrile (HPLC grade) were purchased from Tianjin Kemiou Chemical Reagent Company. Double-distilled water was made in our laboratory.

### 3.2. Methods

#### 3.2.1. Extraction Methods

The dried fruits of *F. suspensa* were crushed and sieved by different standard sieves. Staticultrasonic-assisted extraction was conducted in an ultrasonic bath (Shumei^®^ KQ–5200DE ultrasonic instrument, Kunshan, China) equipped with a digital timer, a water level monitor and a temperature controller. Six sandwich type transducers were uniformly fixed under the bottom of the bath. The bath was a rectangular container with a capacity of 10.0 L (30 × 24 × 15 cm). The frequency and maximum power of the instrument were 40 KHz and 250 W, respectively. The exactly weighted drug powder and solvent were placed into a 100-mL flask, and then, the extraction was performed according to the designed conditions. After extraction, the weight loss was complemented with the solvent.

Maceration extraction was carried out in a thermostatic water bath. The exactly weighted sample powder (1 g, 40–60 mesh) and solvent (20 mL) were accurately added into a clean and dry 100-mL conical flask and then soaked for 10 min. The solvent was the mixture of methanol/water and ethanol/water with different mixed ratios. Heat reflux extraction (HRE) was conducted in a water bath. The exactly weighed drug powder (3 g, 60–80 mesh) was placed into a 100-mL glass flask and extracted with 90 mL 50% aqueous ethanol for 1 and 0.5 h at the boiling point, respectively. Soxhlet extraction (SE) was performed in a Soxhlet apparatus with 1 g accurately weighed drug powder (60–80 mesh). The solvent was methanol (100 mL), and the extraction time was 4 h. Decoction extraction (DE) was performed in a 500 mL glass flask with 10.0 g accurately weighed drug powder and 200 mL distilled water. The extraction was performed three times, and the time was 1.5 h in every cycle. After extraction, the extract was transferred to a volumetric flask for quantitative analysis. All experiments were performed in triplicate.

#### 3.2.2. HPLC Analysis

Forsythiaside A, phillyrin and rutin were quantified by RP–HPLC. A Waters HPLC system equipped with Waters 600 pump, photodiode array detection (PDA) and Empower^TM^ 2 Chromatography Data Software (Waters Technologies, Milford, MA, USA) was used for quantitative analysis. Chromatographic separations were carried out using a Thermo ODS2 column (4.6 mm × 250 mm, 5 μm). The mobile phase consisted of acetonitrile (A) and water with 0.04% (*v*/*v*) phosphoric acid (B). Gradient elution was performed in a linear gradient according to the following program: 0–20 min, 15% A; 21–30 min, 25–30% A; 31–40 min, 30–55% A. The flow rate was 1.0 mL/min. UV detection wavelength was 330, 278 and 350 nm for forsythiaside A, phillyrin and rutin, respectively. Quantitative determination of the active compositions in the extracts was performed using external standards by means of a six-point calibration curve. 

#### 3.2.3. Single Factor Experiments 

The effect of a single factor on UAE including binary mixed solvents of methanol/water and ethanol/water, pH value of the solvent, particle size, temperature, solvent/material ratio, ultrasonic power and extraction time were investigated, respectively.

#### 3.2.4. Response Surface Design

A three-factor (*X*_1_, *X*_2_ and *X*_3_) and three-level (−1, 0 and +1) Box–Behnken response surface design was used to optimize the extraction process. The independent variables were ethanol percentage (*X*_1_, %), solvent to material ratio (*X*_2_, mL/g) and extraction time (*X*_3_, min). The coded and uncoded levels of the independent variables are shown in Table 5. The dependent variables (response variables) were the extraction yields of FSA, PHI and RT (mg/g). The generalized second-order polynomial model proposed for the response surface analysis was as follows:

(1) $$Y=\;\beta_{0}\;+\;\overset{k}{\underset{i\;=1}{\sum }}\beta_{i}X_{i}\;+\;\overset{k}{\underset{i=1}{\sum }}\beta_{ii}X_{i}^{2}+\;\overset{k\;-1}{\underset{i}{\sum }}\overset{k}{\underset{j}{\sum }}\beta_{ij}X_{i}X_{j}\;$$

where *β*_0_, *β_i_*, *β_ii_* and *β_ij_* are regression coefficients for the intercept, linear, quadratic and interaction terms, respectively. *X_i_* and *X_j_* are the coded value of the independent variables, while *k* equals the number of tested factors (*k* = 3).

## 4. Conclusions

The efficient ultrasonic-assisted extraction method was developed for simultaneous extraction of rutin, forsythiaside A and phillyrin from the fruits of *F. suspensa.* The effects of various factors on UAE were investigated in detail. In a certain value range, the factors all showed a significant effect on UAE. The mass transfer mechanism of UAE using different mixed solvents could be further explained by comparison with the maceration extraction method. The response surface methodology was used to optimize the experimental variables including ethanol concentration, solvent to material ratio and extraction time. Compared to conventional extraction methods (HRE, SE and DE), UAE provides the highest extraction efficiency and offers many advantages including the reduction of solvent, temperature and time for extraction. The developed methods would be environmentally-friendly or green process for the simultaneous extraction of the main active components in the fruits of *F. suspensa.* The results would be valuable for explaining the mechanism of UAE of different components from plant materials.

## Figures and Tables

**Table 1:** Box–Behnken design and the responses measured.

No. Exp.	Ethanol Concentration (%)	Solvent Ratio (mL/g)	Extraction Time (min)	Extraction Yield (mg/g)		
				FSA	PHI	RT
1	80	25	10	24.58	2.28	1.66
2	40	15	25	29.14	2.06	1.81
3	80	15	25	23.02	1.96	1.76
4	60	25	25	32.72	2.49	1.87
5	60	35	40	32.80	2.47	1.91
6	60	15	40	30.44	2.19	1.84
7	80	35	25	26.02	2.30	1.74
8	40	25	10	26.42	2.26	1.83
9	60	25	25	32.24	2.48	1.85
10	80	25	40	25.82	2.19	1.80
11	60	25	25	31.26	2.45	1.84
12	60	15	10	29.32	2.2	1.72
13	40	25	40	29.50	2.23	1.97
14	60	25	25	31.5	2.52	1.88
15	40	35	25	31.46	2.34	1.94
16	60	25	25	31.42	2.47	1.86
17	60	35	10	31.68	2.38	1.77

**Table 2:** Estimates of the model coefficients for the responses in UAE.

Coefficient	FSA	PHI	RT
*β*_0_	−9.36	−0.29	1.22
*β*_1_	1.25 **	0.056	7.38 × 10^−3^ **
*β*_2_	0.059 **	0.067 **	0.026 **
*β*_3_	0.32 *	9.28 × 10^−3^	8.94 × 10^−3^ **
*β*_12_	8.50 × 10^−4^	7.50 × 10^−5^	−1.88 × 10^−4^ **
*β*_13_	−1.5 × 10^−3^	−5.00 × 10^−5^	2.08 × 10^−19^
*β*_23_	−2.4 × 10^−17^	1.67 × 10^−4^	3.33 × 10^−5^
*β*_11_	−0.011 **	−4.84 × 10^−4^ **	−5.31 × 10^−5^ *
*β*_22_	3.1 × 10^−4^	−1.24 × 10^−3^ **	−2.63 × 10^−4^ *
*β*_33_	−3.55 × 10^−3^	−2.16 × 10^−4^	−1.06 × 10^−4^ *
Model (*p*-value)	0.0006	0.0002	<0.0001
Lack of Fit (*p*-value)	0.093	0.078	0.358

* *p* < 0.05; ** *p* < 0.01.

**Table 3:** Comparison of UAE with heat reflux extraction (HRE), Soxhlet extraction (SE) and decocting extraction (DE) on the extraction yields of FSA, RT and PHI.

Extraction Methods	Solvent	Extraction Time (min)	Extraction Yields (mg/g)		
			FSA	RT	PHI
UAE	50% Ethanol	37	32.80 ± 1.03	1.90 ± 0.035	2.47 ± 0.11
HRE	50% Ethanol	90	29.44 ± 1.20	1.50 ± 0.030	1.96 ± 0.085
SE	Methanol	240	31.97 ± 0.99	1.74 ± 0.070	2.10 ± 0.067
DE	Water	270	22.15 ± 0.79	1.28 ± 0.065	1.64 ± 0.092

**Table 4:** Regression parameters of HPLC-DAD analysis for the three compounds.

Compounds	Regression Equation	Regression Coefficient (*r*^2^)	Linear Range (µg/mL)
RT	*y* = 2E + 07*x* + 49,211	0.9993	2.30–153.00
FSA	*y* = 2E + 07*x* + 129,820	0.9997	7.40–644.00
PHI	*y* = 1E + 07*x* + 22,223	0.9996	2.06–103.00

**Table 5:** Coded and uncoded levels of the independent variables used for the Box–Behnken design.

Independent Variables	Levels		
	−1	0	1
Ethanol concentration (*X*_1_) (%)	40	60	80
Solvent to material ratio (*X*_2_) (mL/g)	15	25	35
Extraction time (*X*_3_) (min)	10	25	40
